# Comparison of Cefepime with Piperacillin/Tazobactam Treatment in Patients with Hospital-Acquired Pneumonia

**DOI:** 10.3390/antibiotics12060984

**Published:** 2023-05-30

**Authors:** Bo-Guen Kim, Danbee Kang, Kyung Hoon Min, Juhee Cho, Kyeongman Jeon

**Affiliations:** 1 Division of Pulmonary and Critical Care Medicine, Department of Medicine, Samsung Medical Center, Sungkyunkwan University School of Medicine, Seoul 06351, Republic of Korea; kbg1q2w3e@gmail.com; 2 Center for Clinical Epidemiology, Samsung Medical Center, Seoul 06351, Republic of Korea; dbee.kang@skku.edu (D.K.); jcho@skku.edu (J.C.); 3 Department of Clinical Research Design & Evaluation, SAIHST, Sungkyunkawan University, Seoul 06351, Republic of Korea; 4 Division of Pulmonary, Allery, and Critical Care Medicine, Department of Internal Medicine, Korea University Guro Hospital, Seoul 08308, Republic of Korea; minkyunghoon@korea.ac.kr

**Keywords:** healthcare-associated pneumonia, cefepime, piperacillin/tazobactam, drug therapy, treatment outcome

## Abstract

Although cefepime and piperacillin/tazobactam are commonly prescribed for the treatment of hospital-acquired pneumonia (HAP), which one is the superior therapy remains unclear. Using Korean National Health Insurance Service data from January 2018 to December 2018, we compared the clinical outcomes of patients with HAP who were treated with cefepime and those treated with piperacillin/tazobactam. Data from 9955 adult patients with HAP, of whom 1502 (15%) received cefepime and 8453 (85%) received piperacillin/tazobactam, were retrieved for primary analysis. Tube feeding, suctioning, positioning care, and intensive care unit admission were more common among patients who received piperacillin/tazobactam. Treatment outcomes, including rates of in-hospital mortality, pneumonia-related readmission, and all-cause mortality within 6 months after discharge, were comparable between the two groups. In a subgroup analysis of data from patients who required tube feeding, the risk for in-hospital mortality was significantly higher among those who received cefepime (fully adjusted odds ratio, 1.43; 95% confidence interval, 1.04–1.97; *p* = 0.042). Treatment outcomes did not differ between patients who received cefepime and those who received piperacillin/tazobactam treatment, but among patients who were at risk for aspiration, such as those receiving tube feeding, those who received piperacillin/tazobactam had lower rates of in-hospital mortality.

## 1. Introduction

Hospital-acquired pneumonia (HAP) is one of the most common nosocomial infections [1]; its clinical burden is significant, including prolonged hospitalization and increased rates of morbidity and mortality [2]. Although the guidelines for the management of HAP have been updated [3,4], empirical therapy with antibiotics remains a mainstay of treatment. Current guidelines recommend the rapid initiation of broad-spectrum antibiotics, such as cefepime, piperacillin/tazobactam, levofloxacin, and carbapenems, to target common Gram-positive and Gram-negative bacteria [3,4]. However, epidemiological studies have shown a link between increased use of carbapenems and resistance by Gram-negative bacilli (GNB) [5,6,7], which currently represents the greatest threat to patients [8]. Therefore, antimicrobial stewardship to restrict the use of carbapenem has been suggested [9], and initial antimicrobial regimens frequently include two antipseudomonal beta-lactam antibiotics: cefepime (a fourth-generation cephalosporin) and piperacillin/tazobactam (aminopenicillin with beta-lactamase inhibitors). Further selection requires the consideration of several other patient-specific factors.

Although the benefits of cefepime and piperacillin/tazobactam in the management of HAP have been established [3,4], what remains unclear is whether one agent is superior in actual practice [10,11,12]. To determine whether a particular antipseudomonal beta-lactam antibiotic in the initial management of HAP is associated with any benefits, we used national health insurance claims data to compare the clinical outcomes of patients with HAP who received cefepime and those who received piperacillin/tazobactam.

## 2. Methods

### 2.1. Data Source

The Korean National Health Insurance Service (KNHIS) is a public medical insurance system that covers approximately 97% of Koreans. Data used in this study were obtained from the national health claims database established by the KNHIS, which provides robust data about the diagnoses according to the 10th edition of the International Classification of Diseases (ICD-10) and about interventions, prescriptions, and patient demographics [13,14].

### 2.2. Study Design and Population

In this population-based retrospective cohort study, we used data from 1 January 2018 to 31 December 2018 in the KNHIS database. The inclusion criteria were an age of ≥20 years and a diagnosis of HAP during hospitalization for 3 days or more in a tertiary or general hospital, in a manner previously reported [15]. If a patient had multiple in-patient records, we considered only the first episode. We excluded patients who received antibiotic treatments for pneumonia (ICD-10 codes J12–J18, J85.1, and J85.2) within 3 months before the hospitalization. We also excluded patients who were admitted to hospitals via emergency rooms and in whom community-acquired pneumonia was suspected (ICD-10 codes J40, J209, J219, R05, R060, and R509).

To address the primary research objective of comparing clinical outcomes of patients treated with cefepime and those treated with piperacillin/tazobactam for HAP, we arbitrarily defined each antibiotic treatment as at least a 3-day regimen of cefepime or piperacillin/tazobactam during hospitalization. In Korea, the recommended dose for nosocomial pneumonia is 4.5 g of piperacillin/tazobactam every 6 h and 2 g of cefepime every 8 h, respectively. In addition, dose adjustments for antibiotics are generally recommended in patients with impaired renal function to avoid toxicity.

**Figure 1:**
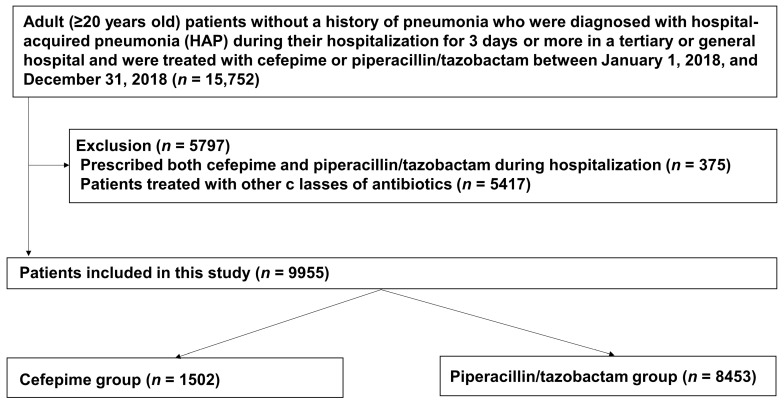
Flowchart of selection of the study population.

During the study period, 15,752 patients with HAP were treated with cefepime or piperacillin/tazobactam. Of those patients, 375 were given both cefepime and piperacillin/tazobactam during the same hospitalization and were therefore excluded from the study. We also excluded 5417 patients who were treated with other classes of antibiotics, such as monobactams, aminoglycosides, glycopeptides, metronidazole, or clindamycin. Since study participants could have both exclusion criteria, a total of 5797 participants were excluded. Data from the remaining 9955 patients treated with either cefepime or piperacillin/tazobactam during hospitalization were retrieved for primary analysis; 1502 patients (15%) had received cefepime, and 8453 (75%) had received piperacillin/tazobactam (Figure 1).

This study was conducted in accordance with the Declaration of Helsinki and approved by the Ethics Committee of Samsung Medical Center (IRB no. SMC201912141-HE002). The requirement for informed consent was waived because of the observational nature of the study and use of only anonymized data.

### 2.3. Data Collection and Clinical Outcomes

Information about sociodemographic characteristics, comorbidities, procedures, prescriptions, and hospital characteristics were collected from the KNHIS data on the basis of claim codes. Comorbidities included cancer, asthma, chronic obstructive pulmonary disease (COPD), other chronic lower respiratory diseases, chronic kidney disease (CKD), end-stage renal disease (ESRD), and anemia, which were defined by ICD-10 codes at admission and within 3 months before hospitalization. Procedures of interest during hospitalization included tube feeding, suctioning, positioning care, mechanical ventilation for more than 3 h, and admission to an intensive care unit (ICU) [15]. Hospitals were classified according to the number of hospital beds and specialties, as defined by Korea’s Medical Service Act [16]. General hospitals are defined as hospitals with more than 100 beds and at least 7 specialty areas, and tertiary hospitals are defined as those with more than 500 beds and more than 20 specialty departments that serve as teaching hospitals for medical students and nurses.

The primary outcome was in-hospital mortality. The secondary outcomes were rehospitalization for pneumonia according to ICD-10 codes J12–J18, J85.1, and J85.2 within 7 days after discharge, and all-cause mortality within 6 months after survival discharge.

### 2.4. Statistical Analysis

We used mixed-effects logistic regression to compare the rates of in-hospital mortality among patients who received cefepime and among those who received piperacillin/tazobactam. Among patients alive at discharge, we conducted the same analysis with rates of readmission for pneumonia within 7 days after discharge. We used multivariable models to estimate odds ratios (ORs) with 95% confidence intervals (CIs). Because patient survival could be clustered by hospital, we used hospitals as a random intercept in the logistic model. In addition, to compare rates of all-cause mortality after discharge by type of antibiotic, the cumulative incidence of clinical events was calculated as a Kaplan–Meier estimate. We used the Cox proportional hazards model to estimate hazard ratios (HRs) for death.

In multivariable models for all the outcomes, we adjusted age, sex, history of hospitalization, comorbidities (including asthma, cancer, COPD, CKD, and ESRD), and location and type of hospital. To adjust for confounding by disease severity, we further adjusted for ICU admission. Furthermore, to minimize confounding effects, we performed sensitivity analyses. First, we used inverse probability treatment-weighted (IPTW) Cox proportional hazards regression to compare group differences in clinical outcomes [17]. All variables were included in this model. Each patient was assigned a weight that was based on the likelihood that he or she received cefepime. Furthermore, we performed stratified analyses to evaluate whether the association of cefepime with clinical outcome differed between patients at and not at risk for aspiration pneumonia, which was defined as use of tube feeding [18].

All *p* values were two-sided, and *p* values of less than 0.05 were considered significant. We used SAS^®^ Visual Analytics (SAS Institute, Cary, NC, USA) and STATA version 17 (StataCorp LLC, College Station, TX, USA) to perform analyses.

## 3. Results

The characteristics of patients with HAP are listed in Table 1. In comparison with the piperacillin/tazobactam recipients, the cefepime recipients were younger; had more comorbidities such as cancer, asthma, and COPD; and were more often admitted to tertiary hospitals. In the procedures of interest during hospitalization, the piperacillin/tazobactam recipients, in comparison with the cefepime recipients, more often received tube feeding (29.1% vs. 23.7%, respectively), suctioning (27.6% vs. 21.8%, respectively), and positioning care (35.6% vs. 26.3%, respectively) and more often were admitted to an ICU (32.5% vs. 26.8%, respectively; Table 1).

CKD, chronic kidney disease; COPD, chronic obstructive pulmonary disease; ESRD, end-stage renal disease; ICU, intensive care unit; SD, standard deviation.

**Figure 2:**
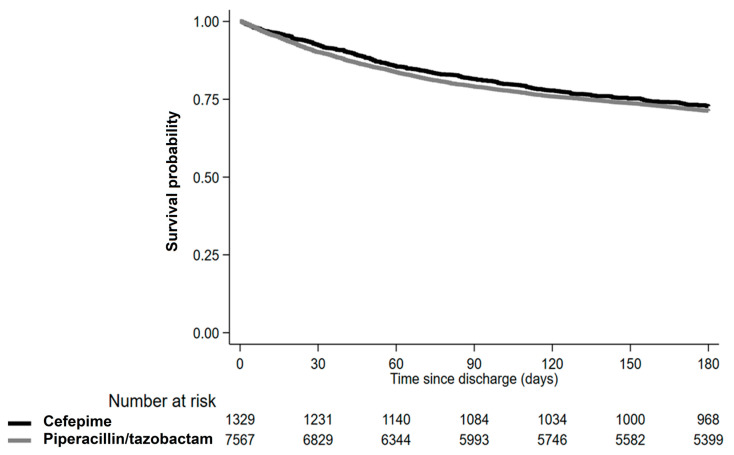
Comparison of rates of all-cause mortality within 6 months after discharge between cefepime and piperacillin/tazobactam recipients.

During hospitalization, 1059 patients died, and in-hospital mortality rates did not differ significantly between the piperacillin/tazobactam recipients and the cefepime recipients (11.5% vs. 10.5%, *p* = 0.230) (Figure 2).

The OR for in-hospital mortality among patients receiving cefepime was 1.05 (95% CI, 0.87–1.27). This association did not change materially after adjustment (adjusted OR, 1.02; 95% CI, 0.85–1.23), and it persisted after adjustment for ICU admission (fully adjusted OR, 1.07; 95% CI, 0.88–1.29; Table 2). IPTW adjustment analysis showed that the OR for in-hospital mortality among cefepime recipients was 1.14 (95% CI, 0.91–1.44). When risk for in-hospital mortality was evaluated according to tube feeding, the association was particularly stronger in cefepime recipients who received tube feeding than in those who did not (fully adjusted OR, 1.43; 95% CI, 1.04–1.97; *p* = 0.042; Table 2).

Of the 8896 patients who were alive at discharge from the hospital, 148 (1.96 per 100 person-years) who received piperacillin/tazobactam and 24 (1.81 per 100 person-years) who received cefepime were rehospitalized for pneumonia within 7 days after discharge. The risk for pneumonia-related readmission was similar in the two groups (fully adjusted OR, 1.05; 95% CI, 0.62–1.78; Table 3), both among patients of both groups who needed tube feeding and among those who did not (Table 3).

With regard to all-cause mortality after discharge, 2538 patients died within 6 months after discharge. The incidence per 100 person-years was 0.20 among the piperacillin/tazobactam recipients and 0.18 among the cefepime recipients (Table 4). The HR for all-cause mortality among the cefepime recipients was 0.95 (95% CI, 0.84–1.08), which was similar to the result of the IPTW adjustment analysis (HR, 0.98; 95% CI, 0.86–1.11; Table 4). The risk for all-cause mortality within 6 months after discharge did not differ between the two groups (Table 4).

## 4. Discussion

In this KNHIS data-based study of the difference in clinical outcomes of patients with HAP treated with cefepime or piperacillin/tazobactam, we found that although tube feeding, suctioning, positioning care, and ICU admission were more common among the piperacillin/tazobactam recipients, the clinical outcomes, including in-hospital mortality, pneumonia-related readmission, and all-cause mortality within 6 months after discharge, were similar in the two groups. However, in a subgroup analysis of patients who required tube feeding the risk for in-hospital mortality was significantly higher among those who received cefepime.

Piperacillin/tazobactam and cefepime are commonly prescribed for the treatment of nosocomial and healthcare-associated infections, not only for their antipseudomonal effect but also for their extended-spectrum activity in empirical therapy [19]. The antimicrobial activity of piperacillin is enhanced by the addition of tazobactam against Gram-positive cocci (GPC), GNB, and anaerobic bacteria; therefore, piperacillin/tazobactam appears to be useful in the treatment of mixed aerobic and anaerobic infections [20]. Cefepime, introduced as a fourth-generation cephalosporin antibiotic, has an extended spectrum of activity against both GPC and GNB. Cefepime is frequently used for the treatment of healthcare-associated infections and shows greater in vitro stability against extended-spectrum beta-lactamase-producing pathogens than do other cephalosporins [21,22]. However, recent guidelines for the management of HAP do not mention superiority of or preference for one of these two antibiotics because evidence is lacking [3,4], although the susceptibility of the majority of GNB to piperacillin/tazobactam and cefepime is better than that to other antibiotics [23].

In previous studies, investigators have compared the use of cefepime and piperacillin/tazobactam in patients with diseases other than HAP. According to Paul et al., who conducted a systematic review and meta-analysis of randomized controlled trials of empirical monotherapy for febrile neutropenia, cefepime was associated with a significantly higher rate of all-cause mortality than were other drugs (imipenem, meropenem, and piperacillin/tazobactam) [24]. In addition, infection-related mortality, bacterial superinfections, and discontinuation of the prescribed treatment were more common with cefepime, whereas no differences in other secondary efficacy outcomes, such as microbiological failure and drug modifications, were observed. Paul et al. also argued that the increased mortality rate observed among patients with febrile neutropenia treated with cefepime should serve as a strong warning against the use of cefepime for that disease. They suggested that ceftazidime, piperacillin/tazobactam, imipenem/cilastatin, and meropenem were suitable as monotherapy for febrile neutropenia. In another single-center retrospective study in the United States, Ross et al. compared the treatment outcomes of cefepime and piperacillin/tazobactam in the initial antibiotic management of patients with septic shock in the ICU [25]. They found no significant differences in common adverse effects but did find a significantly higher mortality rate among patients treated with cefepime. The exact evidence of the higher mortality rate among cefepime recipients in both studies is not clear; therefore, further studies are needed. In a single-center retrospective study in the United States, Luther et al. assessed the outcome of vancomycin therapy in combination with piperacillin/tazobactam or cefepime [26]. Although the study included more than 4000 patients, the authors did not include the treatment outcome for pneumonia alone. They reported that the incidence of acute kidney injury was higher among patients who received vancomycin with piperacillin/tazobactam than among those who received vancomycin with cefepime. However, the in-hospital mortality rate was higher among those treated with vancomycin and cefepime than among those treated with vancomycin and piperacillin/tazobactam, although the difference was not statistically significant.

In our study, we found no significant differences in rates of in-hospital mortality, pneumonia-related readmission, and all-cause mortality within 6 months after discharge between the cefepime and piperacillin/tazobactam recipients. However, the risk for in-hospital mortality and pneumonia-related readmission tended to be higher among patients who received cefepime. This result is consistent with those of previous comparisons of the clinical outcomes after the use of the two antibiotics.

However, the results were different in some cases when it was necessary to cover anaerobes. We performed a subgroup analysis, considering that tube feeding was likely to lead to aspiration pneumonia that necessitated anaerobe coverage [18]. The presence of a nasogastric feeding tube is itself associated with colonization and aspiration of pharyngeal secretions and gastric contents; therefore, the incidence of GNB pneumonia is high among tube-fed patients [27]. The subgroup analysis revealed that the rate of in-hospital mortality was significantly higher among the cefepime recipients than among the piperacillin/tazobactam recipients, and although the rates of pneumonia-related readmission and all-cause mortality did not differ significantly, they tended to be higher among the cefepime recipients. These results suggest that in empirical therapy for HAP in patients at risk for aspiration, such as those receiving tube feeding, piperacillin/tazobactam is preferable because it is a representative antibiotic that could cover most anaerobe bacteria [28]. However, a further prospective study of the clinical benefits of piperacillin/tazobactam in the management of HAP in patients at risk for aspiration is needed.

To the best of our knowledge, this is the first comparison of clinical benefits between piperacillin/tazobactam and cefepime for HAP. However, this study had several potential limitations. First, the definition of HAP according to claim codes has limited accuracy and validity. We tried to use an operational definition of HAP that fitted the definition of existing guidelines [3,4], but that definition might have included misclassifications of HAP. Furthermore, diagnoses based on claim codes can differ from actual clinical diagnoses. However, the KNHIS database is routinely audited, and the data are considered reliable and have been used in numerous peer-reviewed publications [29]. Second, in this study we were not able to evaluate the adverse effects of each antibiotic. In many previous studies of piperacillin/tazobactam, the incidence of nephrotoxicity tended to increase, especially when this combination was used with vancomycin concomitantly [30,31]. Cefepime has been known to be associated with adverse neurologic effects [32,33] and an increased incidence of hospital-acquired Clostridioides difficile infection [34]. The well-known adverse effects of these two types of antibiotics differ, but these effects were not described among the KNHIS data. Because adverse effects of antibiotics can also influence clinicians’ decisions to administer them, a large-scale analysis of adverse effects is needed. Lastly, the results of bacterial etiologies or antibiotic susceptibility tests could not be investigated because such information was not available in KNHIS data. A further large prospective randomized controlled trial taking into account bacterial etiologies and antibiotic susceptibility tests is required to confirm our findings.

In conclusion, we found no significant difference in outcomes of HAP between patients who received piperacillin/tazobactam and those who received cefepime, but among patients at risk for aspiration, such as those receiving tube feeding, the rate of in-hospital mortality was lower among the piperacillin/tazobactam recipients. These results indicate that in selecting the initial empirical antibiotic treatment of HAP, clinicians should understand the characteristics of each antibiotic and the patient’s condition, especially in view of the risk for aspiration.

## Figures and Tables

**Table 1:** Characteristics of study population (N = 9955).

Variables	Piperacillin/Tazobactam (*n* = 8453)	Cefepime (*n* = 1502)	*p* Value
Mean age, years (SD)	74.26 (13.21)	72.14 (13.11)	<0.001
Sex			0.13
Male	5230 (61.87%)	960 (63.91%)	
Female	3223 (38.13%)	542 (36.09%)	
Mean Charlson comorbidity index (SD)	6.21 (3.63)	6.25 (3.44)	0.71
Comorbidity			
Cancer	2370 (28.04%)	526 (35.02%)	<0.001
Asthma	3728 (44.1%)	758 (50.47%)	<0.001
COPD	2269 (26.84%)	503 (33.49%)	<0.001
Other chronic lower respiratory disease	4325 (51.17%)	785 (52.26%)	0.43
CKD	1213 (14.35%)	186 (12.38%)	0.04
ESRD	1216 (14.39%)	188 (12.52%)	0.06
Anemia	2527 (29.89%)	385 (25.63%)	<0.001
History of hospitalization	4041 (47.81%)	759 (50.53%)	0.05
Location of hospital			<0.001
Metropolitan	5889 (69.67%)	1154 (76.83%)	
Rural	2564 (30.33%)	348 (23.17%)	
Type of hospital			<0.001
Tertiary	2341 (27.69%)	577 (38.42%)	
General	6112 (72.31%)	925 (61.58%)	
Tube feeding	2456 (29.05%)	356 (23.7%)	<0.001
Suctioning	2329 (27.55%)	328 (21.84%)	<0.001
Requiring positioning care	3013 (35.64%)	395 (26.3%)	<0.001
Mechanical ventilation	904 (10.69%)	152 (10.12%)	0.50
ICU admission	2748 (32.51%)	403 (26.83%)	<0.001
Co-medication with quinolones	5821 (68.86%)	1015 (67.58%)	0.32

**Table 2:** Odds ratios (95% confidence intervals) for in-hospital mortality according to type of antibiotics (N = 9955).

Population	Piperacillin/Tazobactam	Cefepime
Overall		
Crude	Reference	1.05 (0.87–1.27)
Model 1	Reference	1.02 (0.85–1.23)
Model 2	Reference	1.07 (0.88–1 29)
IPTW analysis	Reference	1.14 (0.91–1.44)
Patients without tube feeding		
Crude	Reference	0.95 (0.75–1.21)
Model 1	Reference	0.91 (0.72–1.15)
Model 2	Reference	0.92 (0.72–1.18)
IPTW analysis	Reference	1.00 (0.78–1.28)
Patients with tube feeding		
Crude	Reference	1.37 (1.01–1.87)
Model 1	Reference	1.39 (1.01–1.91)
Model 2	Reference	1.43 (1.04–1.97)
IPTW analysis	Reference	1.49 (1.05–2.11)

IPTW, inverse probability treatment-weighted. Model 1: Stratified by hospital and adjusted for age, sex, in-patient history, asthma, cancer, chronic kidney disease, end-stage renal disease, location of hospital, and type of hospital. Model 2: Further adjusted for intensive care unit admission.

**Table 3:** Odds ratios (95% confidence intervals) for readmission for pneumonia within 7 days after discharge according to type of antibiotics (N = 8896).

Population	Piperacillin/Tazobactam	Cefepime
Overall		
Crude	Reference	1.00 (0.59–1.70)
Model 1	Reference	1.06 (0.63–1.80)
Model 2	Reference	1.05 (0.62–1.78)
IPTW analysis	Reference	1.13 (0.66–1.95)
Patients without tube feeding		
Crude	Reference	0.90 (0.49–1.64)
Model 1	Reference	0.95 (0.52–1.72)
Model 2	Reference	0.94 (0.52–1.71)
IPTW analysis	Reference	1.07 (0.58–1.94)
Patients with tube feeding		
Crude	Reference	1.36 (0.52–3.55)
Model 1	Reference	1.44 (0.55–3.78)
Model 2	Reference	1.42 (0.54–3.74)
IPTW analysis	Reference	1.34 (0.50–3.63)

Model 1: Stratified by hospital and adjusted for age, sex, in-patient history, asthma, cancer, chronic kidney disease, end-stage renal disease, location of hospital, and type of hospital. Model 2: Further adjusted for intensive care unit admission.

**Table 4:** Hazard ratios (95% confidence intervals) for all-cause mortality within 6 months after discharge according to type of antibiotics (N = 8896).

Population	Piperacillin/Tazobactam	Cefepime
Overall		
Number of deaths	2176	362
Incidence per 100 person-years	0.20	0.18
Crude	Reference	0.92 (0.82–1.05)
Model 1	Reference	0.92 (0.81–1.05)
Model 2	Reference	0.95 (0.84–1.08)
IPTW analysis	Reference	0.98 (0.86–1.11)
Patients without tube feeding		
Number of deaths	1351	242
Incidence per 100 person-years	0.16	0.15
Crude	Reference	0.93 (0.80–1.07)
Model 1	Reference	0.91 (0.79–1.06)
Model 2	Reference	0.92 (0.80–1.07)
IPTW	Reference	0.95 (0.81–1.10)
Patients with tube feeding		
Number of deaths	825	120
Incidence per 100 person-years	0.29	0.32
Crude	Reference	1.11 (0.91–1.37)
Model 1	Reference	1.14 (0.93–1.40)
Model 2	Reference	1.15 (0.93–1.42)
IPTW analysis	Reference	1.18 (0.97–1.44)

Model 1: Stratified by hospital and adjusted for age, sex, in-patient history, asthma, cancer, chronic kidney disease, end-stage renal disease, location of hospital, and type of hospital. Model 2: Further adjusted for intensive care unit admission.

## Data Availability

The data that support the findings of this study are available from the Korean National Health Insurance Service (KNHIS) database. KNHIS data are available for researchers who meet the criteria for access to confidential data. Data access information is available at https://nhiss.nhis.or.kr/bd/ab/bdaba000eng.do (accessed on 11 March 2023). However, data analyzed for this study cannot be shared on any publicly available repository because of legal and confidentiality requirements.
